# The Institutional Worker

**Published:** 1911-12-30

**Authors:** 


### INSTITUTIONAL WORKER

**Being a Special Supplement to "The Hospital"**

### Editor's Notices.

**Contributions:**

Contributions should be written, or preferably typed, on one side of the paper only, and all articles sent in are accepted upon the distinct understanding that they are forwarded to The Hospital only.

The Editor cannot undertake to return MSS. not used, but when a stamped directed envelope is enclosed the MSS. may be returned if a special request is made.

Accepted articles and paragraphs of news will be paid for after publication at the scale rate.

**Address:**

To prevent delay all contributions and letters on editorial business must be addressed exclusively to the Editor, "The Hospital," 29 Southampton Street, Strand, London, W.C. It is important that this regulation shall be strictly observed.

**Correspondence:**

Correspondence on all subjects is invited. The name and address of correspondents must be given as a guarantee of good faith, but not necessarily for publication.

**Special Articles:**

Special articles are invited, and questions, inquiries, and paragraphs upon all matters relating to the work, administration, and management of general, special, mental, fever, cottage and convalescent hospitals, sanatoria, homes, institutions, societies and organisations for the treatment or care of the sick, injured and dependents of all classes. Every matter affecting the interests and welfare of the staff of all grades working in these institutions will receive special consideration.

**Administrative Medicine, Research and Items of News:**

Special terms will be paid for approved articles on subjects in this wide field by experts who have made some section of it their special study and interest. We wish to give prominence to every movement tending to advance accuracy of diagnosis, efficiency in treatment, and the development of modern methods to eradicate disease and promote the welfare of the suffering.

**A Bureau of Information:**

We invite inquiries and applications for information and help in providing for that numerous class of sufferers who require special care or house-room which they cannot obtain in their own homes or secure for themselves. We cannot, however, prescribe or recommend practitioners.

**Books for Review:**

Publishers are particularly requested to send advance proofs of any new books of importance whenever possible, as the Editor has made arrangements to publish immediate reviews on a new plan.

**Photographs, Plans, Blocks, and Illustrations:**

It is requested that wherever possible MSS. may be accompanied by illustrations in any of the above forms. The name of the sender and of the article to which it belongs should be written on the back of each photograph, drawing, or block for purposes of identification.

**Local Papers:**

Newspapers containing reports or news paragraphs should be marked and addressed to the Sub-Editor.

**The Coupon System:**

A coupon will be found at the bottom of the third inside page of the cover each week. This coupon must be attached to every question or inquiry to which an answer is desired in The Hospital.

### Manager's Notices.

Letters relating to the publication, sale, and advertising departments of The Hospital must be addressed to the Manager, "The Hospital," The Hospital Building, 28 and 29 Southampton Street, Strand, London, W.C.

**Subscriptions** which may begin at any time, are payable in advance. Cheques and Post-Office orders, crossed London County and Westminster Bank, Covent Garden branch, should be made payable to the Manager of The Hospital and addressed to him at The Hospital Building, 28 and 29 Southampton Street, Strand, London, W.C.

**Advertisements:**

All advertisements for The Hospital must reach the Manager not later than Wednesday morning in each week. For scale of charges see pages v and 2.

**Classified Advertisements:**

All small advertisements will be classified, for this section of the paper is widely read and of special interest. We wish to encourage those wanting appointments who are attracted to institutional work, and therefore offer them the reduced rate of *twenty-one words and under for* 1s., and *for every ten and part of ten words beyond*, 6*d*.

### POINTS FOR INSTITUTION AUTHORITIES.

The economical management of an Institution depends very greatly upon purchasing supplies in the cheapest market.

It is often impossible locally to secure the highest quality at the lowest prices.

In consequence, a section will be reserved in this part of the paper for announcements of Institutions inviting Tenders foı Contracts.

This will enable Authorities of Institutions to secure tenders for supplies from all over the country, and ensure purchases at the lowest possible prices consistent with quality.

The charge for Tenders is 6s. per inch, and for this small outlay many pounds may be saved on supplies of all descriptions.

**Special Rates** will be quoted for those institutions which agree to send all their vacancy, contract, and public notices to The Hospital each year, so as to make it a file of such announcements for ready reference.

### POINTS FOR TRADE ADVERTISERS.

The Hospital is the organ of Institutions, whether of a Voluntary Character or those under Local Government, Poor Law and Municipal Authorities.

It is read by all Executive Officials and those responsible for the Construction, Management, and Equipment of Public Establishments.

It is also the Journal of the worker engaged in the Medical, Health and Preventive services.

There is no medium more appropriate and effective in the promotion of business in these directions than The Hospital.

### POINTS FOR INSTITUTIONAL WORKERS.

A very large number of Appointments Vacant appear every week in the columns of The Hospital.

If you cannot find a position suited to your requirements, make your wishes known through our Situations Wanted Column.

### Miscellaneous.

ACCOUNTANCY. — BOOKS POSTED and checked, PROFIT AND LOSS ACCOUNTS prepared, BOOK DEBTS collected. AUDITS, CREDITORS arranged with, by Mr. J. C. DACIE, Accountant (33 years in the Wholesale Trade), at 37 King William Street, London Bridge, E.C. Tel. 9488 City. (5279)

### Midwifery.

### APPOINTMENTS VACANT.

### Poor-Law Vacancies.

### Appointments Vacant.

ASSISTANT LABOUR MASTER (£30), Bethnal Green Guardians. Mr. D. Thomas, C. to G., Bishops Road, Victoria Park, N.E.
ASSISTANT MASTER (£30), Burton-on-Trent Union. Mr. C. F. Chamberlin, C. to G., Burton-on-Trent. Dec. 30.
ASSISTANT NURSE (£30), Cuckfield Union. Mr. E. J. Waugh, C. to G., Hayward's Heath. Jan. 4.
ASSISTANT NURSE (£25), Bedford Union. Mr. W. Payne, C. to G., Bedford. Jan. 8.
ASSISTANT NURSE (£22), St. Albans Union. Mr. R. W. Brabant, C. to G., St. Albans.
ASSISTANT NURSE (£25), Evesham Union. E. H. Wadams, C. to G., Union Offices, Evesham. Jan. 6.
ASSISTANT NURSES (£22), Sevenoaks Union. Mr. F. H. Vibart, C. to G., Bank Chambers, Sevenoaks. Jan. 1.
ASSISTANT NURSES (£26), Huddersfield Union. Mr. E. A. Rigby, C. to G., Huddersfield. Jan. 11.
ATTENDANT AND ASSISTANT LABOUR MASTER (£35), King's Norton Union. Mr. R. J. Curtis, C. to G., Guildhall Buildings, Birmingham.
CHARGE AND MIDWIFERY NURSE (£32), Northampton Union. W. Fawkes, C. to G., 4 Derngate, Northampton. Jan. 10.
CHARGE NURSE (£30), Richmond Union. Mr. P. Umney, C. to G., Parkshot, Richmond, Surrey. Jan. 10.
CHARGE NURSE (£30), Guildford Union. Mr. W. S. V. Cullerne, C. to G., Guildford. Jan. 5.
CHARGE NURSE (£30), Mutford and Lothingland Union. Mr. F. Peskett, C. to G., Lowestoft.
CHILDREN'S ATTENDANT (£24), Newport Union. Mr. A. H. Rees, C. to G., Newport, Mon. Jan. 10.
FEMALE IMBECILE ATTENDANT (£26), Willesden Guardians. Mr. J. Hutton Haylor, C. to G., 357 High Road, Brondesbury, N.W. Jan. 3.
FEMALE ASSISTANT EPILEPTIC WARD ATTENDANT (£18), Holborn Union. Matron of the Workhouse, Mitcham, Surrey.
FOSTER-MOTHER (£30), Burnley Union. Mr. J. S. Horn, C. to G., Burnley. Jan. 3.
FOSTER-MOTHER (£28), Greenwich Union. Mr. S. Saw, C. to G., East Greenwich. Jan. 4.
FOSTER-MOTHER (£25), Horsham Union. Mr. A. C. Coole, C. to G., Horsham. Dec. 30.
FOSTER-MOTHER (£25), Bishop Stortford Union. Mr. A. G. Gwynn, C. to G., Bishop Stortford. Jan. 6.
FOSTER-MOTHER (£24), Meriden Union. Mr. A. W. Liggins, C. to G., 11 Priory Street, Coventry. Jan. 11.
HEAD NURSE (£45), East Preston Union. Mr. A. Shelley, C. to G., Littlehampton. Jan. 13.
HOUSEMOTHER (£24), Southwark Union. Mr. S. Wood, C. to G., Ufford Street, Blackfriars, S.E. Jan. 4.
INDUSTRIAL TRAINER AND FOSTER-MOTHER (married couple) (£35 and £30), Kingston Union. Mr. C. W. Dash, C. to G., Kingston-on-Thames.
INFANTS' ATTENDANT (£25), Darlington Union. Mr. C. H. Leach, C. to G., Darlington. Jan. 6.
LABOUR MASTER (£32), York Union. Mr. G. Sykes, C. to G., York. Jan. 2.
MALE ATTENDANT (£28), Bristol Guardians. Mr. J. J. Simpson, C. to G., Bristol. Dec. 30.
MALE ATTENDANT (£30), Devonport Guardians. Mr. A. Gard, C. to G., Devonport. Dec. 30.
MALE LUNATIC ATTENDANT (£30), Hartlepool Union. Mr. G. Kilvington, C. to G., West Hartlepool. Jan. 2.
MASTER AND MATRON (£100), Grimsby Union. Mr. J. F. Wintringham, C. to G., Great Grimsby. Jan. 1.
MATRON FOR SCATTERED HOMES (£50), Guildford Union. Mr. W. S. V. Cullerne, C. to G., Guildford. Jan. 5.
MIDWIFE (£35), Marylebone Guardians. Matron of the Workhouse, Northumberland Street, W.
MIDWIFE (£40), Holborn Union. Matron of the Workhouse, 1A Shepherdess Walk, N.
NIGHT SISTER (£40), Brighton Guardians. Mr. B. Burfield, C. to G., Parochial Offices, Brighton. Jan. 2.
NIGHT SISTER and ASSISTANT SUPERINTENDENT NURSE (£36), Newport (Mon.) Union. A. H. Rees, C. to G., Union Offices, Queen's Hill, Newport (Mon.). Jan. 10.
NIGHT SUPERINTENDENT (£40), Ecclesall Bierlow Union. J. E. Moulding, C. to G. Union Offices, The Edge, Sheffield. Jan. 2.
NIGHT SUPERINTENDENT and MASSEUSE (£38), Brentford Union. Mr. W. Stephens, C. to G., Union Offices, Isleworth, W. Jan. 2.
NURSE (£20), St. Giles and Bloomsbury Guardians. Mr. J. Appleton, C. to G., Broad Street, Bloomsbury, W.C. Dec. 30.
NURSE (£26), Hastings Union. Mr. A. R. Inskipp, C. to G., Hastings. Jan. 1.
NURSE (£30), Hayfield Union. Mr. A. Walker, C. to G., New Mills, near Stockport. Jan. 2.
NURSES (£30), Gateshead Union. G. Craighill, C. to G. Poor Law Union Offices, Gateshead. January 6.
NURSES (£28), Newton Abbot Union. Mr. F. Horner, C. to G., Newton Abbot. Jan. 9.
NURSES (2) (£31 11s.), East Preston Union. Mr. A. Shelley, C. to G., Littlehampton. Jan. 13.
NURSES (£23), Greenwich Union. Mr. S. Saw, C. to G., East Greenwich.
PROBATIONER (£10), North Bierley Union. Mr. W. G. Cooper, C. to G., 4 Town Hall Street, Bradford. Dec. 30.
PROBATIONER (£10), Bromyard Union. Mr. F. W. Nicholl, C. to G., Bromyard. Jan. 1.
PROBATIONER NURSE (£5), Mansfield Union. W. S. Cockerham, C. to G., 105 Stockwell Gate, Mansfield. Dec. 30.
SISTER (£35), Medway Union. A. Reynolds Norman, C. to G., 22 High Street, Chatham. Jan. 16.
STAFF NURSE (£30), Kidderminster Union. Mr. F. E. Burcher, C. to G., Kidderminster. Jan. 8.
STAFF NURSE (£25), Winchester Union. Mr. F. Faithfull, C. to G., Winchester. Jan. 8.
STAFF NURSE (£24), Holborn Union. Matron of the Workhouse, 1A Shepherdess Walk, N.
SUPERINTENDENT NURSE (£50), Gloucester Union. Mr. H. A. Armitage, C. to G., Gloucester. Jan. 2.
SUPERINTENDENT NURSE (£30), Runcorn Union. Mr. G. F. Ashton, C. to G., Runcorn. Jan. 2.

Superintendent Nurse (£38), Bedford Union. Mr. W. Payne, C. to G., Bedford. Jan. 8.
Ward Sister (£30), Bermondsey Guardians. Matron, the Infirmary, Lower Road, Rotherhithe, S.E.

### Situations Vacant.

Assistant Cook (£20), Hammersmith Guardians. Matron of the Infirmary, Wormwood Scrubbs, W.
Assistant Cook (£20), Paddington Guardians. Matron of the Infirmary, 285 Harrow Road, W.
Barber and Relief Officer (£30), Wolstanton and Burslem Union. Mr. J. E. Lowndes, C. to G., Burslem. Jan. 2.
Cook (£20), Gainsborough Union. Mr. D. M. Robbs, C. to G., Gainsborough. Dec. 30.
Cook (£35), Derby Union. Mr. N. Twigge, C. to G., Derby. Jan. 4.
Cook (12s. weekly), Bermondsey Guardians. Matron of the Workhouse, Parish Street, Tooley Street, S.E.
Engineer (35s. weekly), East Preston Union. Mr. A. Shelley, C. to G., Littlehampton. Dec. 30.
Laundress (£30), Guildford Union. Mr. W. S. V. Cullerne, C. to G., Guildford. Jan. 5.
Laundress (£25), Bishop Stortford Union. Mr. A. G. Gwynn, C. to G., Bishop Stortford. Jan. 6.
Laundress (£25), Bosmere and Claydon Union. Mrs. Cheeseman, Matron of the Hospital, Barham, near Ipswich.
Laundress (£27 10s.), Thetford Union. Mr. J. Houchen, C. to G., Thetford.
Male Cook (£40), Leeds Union. Mr. J. H. Ford, C. to G., Leeds. Dec. 30.

### Institutional and Administrative Vacancies.

### Appointments Vacant.

Lady Superintendent (£65), West Kent General Hospital, Maidstone. Secretary. Jan. 15.
Matron (£60), Putney Hospital. Secretary, 198 Upper Richmond Road, Putney, S.W. Dec. 31.
Matron (£63), Royal Albert Hospital, Devonport. Captain C. W. Dickinson, R.N., Secretary. Jan. 10.
Matron, Jessop Hospital for Women, Sheffield. J. Henderson, secretary, 6 East Parade, Sheffield.
Sister (£30), Queen's Hospital, Birmingham. Apply, Matron. Jan. 7.

### Municipal Vacancies.

### Appointments Vacant.

Assistant Estate Agent (£150), Swansea T.C. Mr. H. L. Coath, Town Clerk, Swansea. Jan. 2.
Assistant Inspector of Nuisances and Health Visitor (£100), Borough of Stafford. Mr. R. Battle, Town Clerk, Borough Hall, Stafford. Jan. 8.
Assistant Nurse (£25). Cheadle R.D. Council Isolation Hospital. F. S. Cox, clerk, Cheadle, Stoke-on-Trent. Jan. 2.
Chief Constable (£350), Lincoln T.C. Mr. W. T. Page, Town Clerk, Lincoln. Jan. 6.
Clerk (£52), Broadstairs and St. Peter's U.D.C. Mr. L. A. Skinner, Clerk to the Council, Broadstairs. Jan. 6.
Clerk of Works (Sewers) (£3 3s. weekly), Salford T.C. The Town Clerk, Salford. Jan. 8.
District Sanitary Inspector (35s. weekly), Stockport T.C. Dr. H. E. Corbin, Medical Officer of Health, Stockport. Dec. 30.
Engineering Assistant (£110), Birkenhead T.C. Mr. C. Brownridge, Borough Engineer, Birkenhead. Jan. 4.
Inspector of Nuisances (£100), Dorking R.D.C. Mr. W. J. Down, Clerk to the Council, Dorking. Jan. 4.
Inspector of Nuisances (£110), Thirsk R.D.C. Mr. W. Swarbreck, Clerk to the Council, Thirsk. Jan. 8.
Inspector of Nuisances (£120), Hemsworth R.D.C. Mr. J. Scholefield, Clerk to the Council, Hemsworth, near Wakefield. Jan. 2.
Nurse (£30), Weston-super-Mare U.D. Council. S. C. Smith, Clerk, Town Hall, Weston-super-Mare. Jan. 2.
Nurse (£36), Epsom U.D. Council Isolation Hospital. E. G. Wilson, Clerk, Church Street, Epsom. Jan. 15.
Rate Clerk (£105), Hackney B.C. The Town Clerk, Hackney, N.E. Jan. 1.
Sanitary Inspector (£120), Haslingden T.C. Mr. W. Musgrove, Town Clerk, Haslingden. Jan. 2.
Superintendents of Working-class Dwellings (2) (£2 10s. and £2 2s.), L.C.C. The Clerk to the Council, Spring Gardens, S.W. Jan. 8.
Surveyor and Engineer, etc. (£100), Warminster R.D.C. Mr. T. Ponting, Clerk to the Council, Warminster. Jan. 6.
Temporary Nurses (two), £35. Kingston-upon-Hull Corporation Isolation Hospital. Apply, Medical Officer of Health, Town Hall, Hull. Jan. 8.
Town Clerk (£500), Taunton T.C. Mr. G. H. Kite, Town Clerk, Taunton. Dec. 31.
Town Clerk (£850), Wandsworth B.C. Mr. H. G. Hills, Town Clerk, Wandsworth, S.W. Jan. 8.

### Miscellaneous Vacancies.

### Appointments Vacant.

Assistant Matron (£40), Dykebar Asylum, Paisley. Apply, Medical Superintendent.
Health Visitor and School Nurse (£104), Leicestershire County Council. W. J. Freer, Clerk, 10 New Street, Leicester. Jan. 2.
Railway Supervisor (£400), Port of London Authority. The Secretary, 109 Leadenhall Street, E.C. Dec. 30.
Veterinary Inspector, Wilts C.C. Mr. R. W. Merriman, Clerk to the Council, Trowbridge. Dec. 31.

### APPOINTMENTS, RESIGNATIONS, &c.

### Poor Law Appointments.

Bambridge, Miss F., Assistant Schoolmistress at the Enfield School, Edmonton Union.
Brough, Miss M., Superintendent Nurse, Leigh Union.
Hoare, Miss R., Matron of the Workhouse, Gower Union.
Holland, Miss M. H., Superintendent Nurse, Whitechapel Union.
Williams, Mr. W. M., Master of the Workhouse, Gower Union.

Charge Nurses.

Ash, Miss M., Lambeth Parish.
Day, Miss M. E., Isle of Wight Union.
Handley, Miss E., Southampton Union.
Mair, Miss A., Lambeth Parish.
Widger, Miss A. E., Southampton Union.
Yorke, Mrs. E. A., Hartlepool Union.

Nurses.

Anderson, Miss A. L., Southwark Union.
Archer, Miss E. M., Poplar and Stepney Sick Asylum District.
Atkinson, Miss M., Winchester Union.
Brudenell, Miss M. E., Poplar and Stepney Sick Asylum District.
Cooper, Mr. W., Keighley Union.
Goodlett, Miss A. C., Poplar and Stepney Sick Asylum District.
Lightley, Miss M. A., West Ashford Union.

